# Long-Term Exposure to Urban Particulate Matter on the Ocular Surface and the Incidence of Deleterious Changes in the Cornea, Conjunctiva and Retina in Rats

**DOI:** 10.3390/ijms21144976

**Published:** 2020-07-14

**Authors:** Wan Seok Kang, Hakjoon Choi, Goeun Jang, Ki Hoon Lee, Eun Kim, Kyeong Jo Kim, Gil-Yeon Jeong, Jin Seok Kim, Chang-Su Na, Sunoh Kim

**Affiliations:** 1 Central R&D Center, Bioresources and Technology (B&Tech) Co., Ltd., Gwangju 61239, Korea; kws2602@hanmail.net (W.S.K.); ohchj12@naver.com (H.C.); goeun2748@gmail.com (G.J.); leekh7@epost.kr (K.H.L.); rubsang84@gmail.com (E.K.); kkkjzzang@nate.com (K.J.K.); gyjeong90@nate.com (G.-Y.J.); keki2000@naver.com (J.S.K.); 2 College of Korean Medicine, Dongshin University, 185 Geonjae-ro, Naju-si, Jeollanam-do 58245, Korea; nakugi@daum.net

**Keywords:** urban particulate matter, cornea, conjunctiva, retina, apoptosis, ocular pressure

## Abstract

We investigated the time-dependent deleterious ocular changes induced by urban particulate matter (UPM) in vitro and in vivo. UPM treatment decreased human corneal epithelial cell migration and survival. Fluorescein scores were consistently increased by UPM application for 16 weeks. One week of rest at 2 or 4 weeks led to a recovery trend, whereas two weeks of rest at 8 weeks induced no change. UPM treatment decreased the tear film break-up time at 2 weeks, which was thereafter maintained until 16 weeks. No changes were found after periods of rest. UPM-treated eyes exhibited greater corneal epithelium thickness than normal eyes at 2 weeks, which recovered to normal at 4 and 8 weeks and was significantly decreased at 16 weeks. Apoptotic cell number in the epithelium was increased at 2 weeks, which remained constant except at 8 weeks. IL-6 expression in the cornea of the right eye continually increased for 16 weeks, and significant recovery was only observed at 8 weeks after 2 weeks of rest. Ocular pressure was significantly increased in the right eye at 12 and 16 weeks. Topical UPM application to the eye induced deleterious changes to various closely related parts of the eye.

## 1. Introduction

Air pollution has severe adverse effects on health that have become more pronounced in recent years. The eye is one of the organs vulnerable to environmental risk because it is constantly exposed to the external environment. Harmful effects of air pollution on the eye, such as eye irritation, blepharitis and conjunctivitis, are well reported [1,2,3]. Recently, increasing evidence has suggested that particulate matter (PM) is a key component exacerbating ocular diseases. PM is composed of various solid and liquid particles that endanger many organs, including the eyes, skin and even the cardiovascular, cerebrovascular and respiratory systems [4,5,6,7]. According to its aerodynamic diameter, PM is divided into PM10 (particle size between 2.5 μm and 10 μm) and PM2.5 (size less than 2.5 μm), and the effects of each size of PM on health have been actively investigated. In addition, the increase in the number of patients with allergic conjunctivitis during periods of higher levels of PM indicates the obvious relationship between PM and eye diseases [8,9]. To date, since most studies on PM and the eye have been limited to in vitro cell models that investigate short-term effects or indirect evidence, extensive, long-term investigations of the effects of PM on the eye are required.

Two studies published by one group examined the effects of PM2.5 or PM10 acquired from a city and demonstrated that both types of PMs similarly induced negative changes, such as increased injury on the corneal surface and reduced tear volume, upon treatment with the same amount of PMs by the same method in mice [10,11]. A human study also demonstrated that people exposed to severe air pollution can suffer symptoms such as dry eye syndrome, lowered tear volume and tear break-up time (TBUT) [12]. Therefore, the data of all these studies also suggest that both PM2.5 and PM10 cause dry eye-like symptoms. An in vitro study using human corneal cells suggested that diesel exhaust particles (DEPs) increase cytotoxicity and inflammatory and oxidative stress responses [13], while other studies have suggested that PM2.5 collected from cities inhibited cell migration [14] and induced autophagy [15].

As referenced here, the deleterious effects of PM directly affecting ocular health are obvious, but the sources of PM widely differ. Researchers investigating the effects of PM frequently disregard the differences in the particle composition of PM [16]. However, some studies have suggested that the compositions of components are more important than the concentrations of PM in terms of toxic effects [17]. A study using Standard Reference Material (SRM 2786) showed that the long-term effects of PM2.5 treatment for 6 months in mice also result in PM2.5-induced dry eye-like symptoms, but the results did not clarify the differences between long-term and short-term exposure [18].

Additionally, most studies investigating the effects of PM have focused on corneal surface and conjunctival changes, but few studies have investigated retinal changes by PM exposure. A human study suggested that PM2.5 does not change intraocular pressure (IOP) but increases self-reported glaucoma and thins the ganglion cell–inner plexiform layer (GCIPL) [19]. There are no reports researching the effects of PM on the retina in animal models.

Therefore, the aim of this study was to explore the changes in the cornea, the conjunctiva and the retina by applying urban particulate matter (UPM) as an eye drop during the short term to the long term. In particular, we sought to clarify the effects of UPM for long-term applications, and the mechanisms were explored.

## 2. Results

### 2.1. Effects of UPM on the Migration and Survival of HCE Cells

**Figure 1:**
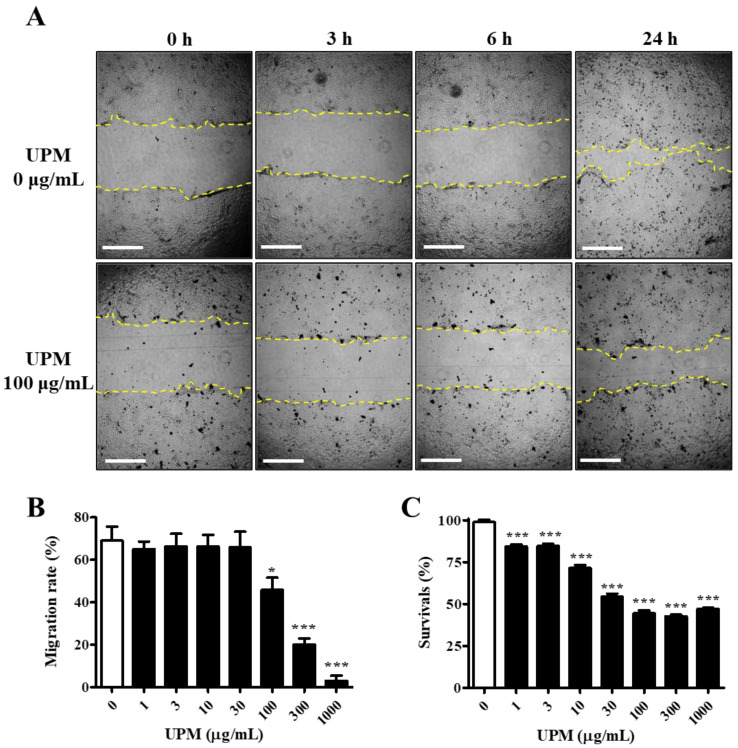
Effect of UPM on the migration activities of human corneal epithelial (HCE) cells. (**A**) HCE cells treated with UPM at various concentrations for 24 h were scratched, and the empty area (yellow lines) was measured. Each area was measured after scratching at 3, 6 and 24 h. The scale bars indicate 100 μm. (**B**) The relative migration rate of HCE cells at 24 h is expressed as the mean ± SD. (**C**) The survival rate of HCE cells treated with UPM at various concentrations for 24 h is expressed as the mean ± SD. * *p* < 0.05, *** *p* < 0.001 compared with 0 μg/mL UPM.

The UPM used in this study consisted of various sizes of particles, and the mean particle diameter was 5.85 μm. The migration activity was measured by a scratch wound healing assay. The cells were exposed to various concentrations of UPM for 24 h, and scratches were induced. The migration rate was not affected by less than 100 μg/mL UPM, but it rapidly decreased by 34% upon exposure to 100 μg/mL UPM and reached 3.0% at the highest concentrations (Figure 1A,B). However, cell survival was dose-dependently decreased and reached 47.2% by UPM (Figure 1C). Therefore, UPM decreased the migration activity and survival of human corneal epithelial (HCE) cells.

### 2.2. Effects of UPM on the Ocular Surface in Rats

**Figure 2:**
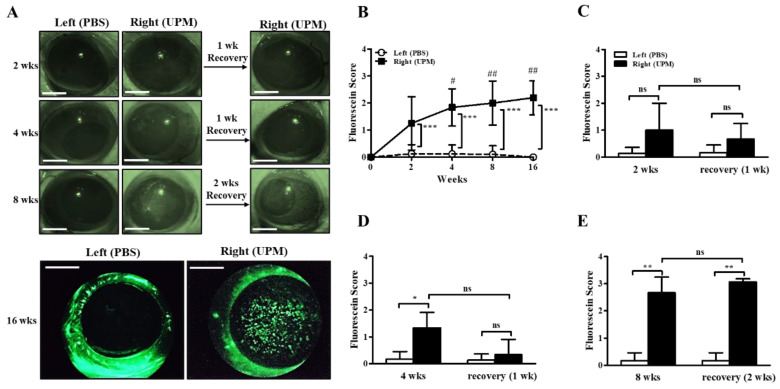
Effect of UPM on the corneal surface according to exposure period. Injury to the corneal surface was determined by fluorescein staining, and its levels were expressed as scores. (**A**) Photographs of fluorescein-stained eyes acquired at 2, 4, 8 and 16 weeks after applying UPM or PBS to the right or left eye. Additional photographs were also acquired after one or two weeks of rest at 2, 4 and 8 weeks. The scale bars indicate 2 mm. (**B**) Fluorescein scores were determined from images at 2, 4, 8 and 16 weeks. Fluorescein scores after one week of rest at 2 weeks (**C**) or 4 weeks (**D**) and after two weeks of rest at 8 weeks (**E**) are shown. Data are expressed as the mean ± SD. * *p* < 0.05, ** *p* < 0.01, *** *p* < 0.001 vs. left eye at the same time point; # *p* < 0.05, ## *p* < 0.01 vs. right eye at 2 weeks; ns, not significant.

The concentration of UPM for topical application to the eye was determined according to previous studies [10,11,18]. Injury of the ocular surface was assessed by scoring fluorescein staining levels on the cornea. The fluorescein scores of the right eye were increased by 2 weeks of UPM exposure compared with the left, untreated eye, and the fluorescein scores were consistently increased until 16 weeks (Figure 2A,B). In addition, the scores at 4, 8 and 16 weeks were significantly higher than the scores at 2 weeks. One week of rest at 2 or 4 weeks led to a trend toward recovery without significance, but two weeks of rest at 8 weeks had no affect (Figure 2C–E). Therefore, the results demonstrated that long-term exposure to UPM constantly increased ocular surface damage and was not easily recovered.

### 2.3. Effects of UPM on the Stability of Tear Film and Tear Volume

**Figure 3:**
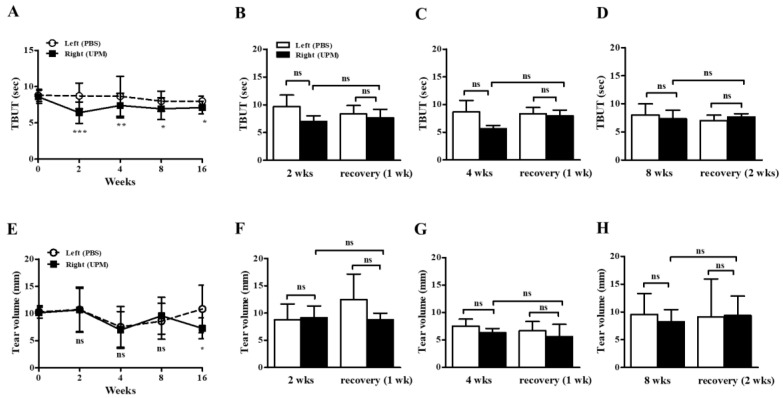
Effect of UPM on TBUT and tear volume according to exposure period. The TBUT values were measured at 2, 4, 8 and 16 weeks (**A**), after one week of rest one at 2 weeks (**B**) or 4 weeks (**C**), and after two weeks of rest at 8 weeks (**D**). The wet lengths of thread were expressed as tear volume measured at 2, 4, 8 and 16 weeks (**E**), after one week of rest at 2 weeks (**F**) or 4 weeks (**G**), and after two weeks of rest at 8 weeks (**H**). Data are expressed as the mean ± SD. * *p* < 0.05, ** *p* < 0.01, *** *p* < 0.001 vs. left eye at the same time point; ns, not significant.

At day 0, there were no significant differences in tear film break-up time (TBUT) or tear volume between the left (phosphate buffered saline, PBS) and right (UPM) eyes. From 2 weeks after the beginning of UPM exposure, TBUTs were similarly decreased at all time points (Figure 3A). One or two weeks of rest at 2, 4 and 8 weeks did not lead to any recovery trend (Figure 3B–D). On the other hand, tear volume was significantly decreased during the last 16 weeks in the right eye (Figure 3E). There was no trend in the changes by rest (Figure 3F–H). Therefore, the stability of tear film was decreased after 2 weeks of exposure to UPM but was not severely changed by long-term exposure, and a decrease in tear volume was only found after long-term exposure.

### 2.4. Effects of UPM on Corneal Thickness and Cell Apoptosis

**Figure 4:**
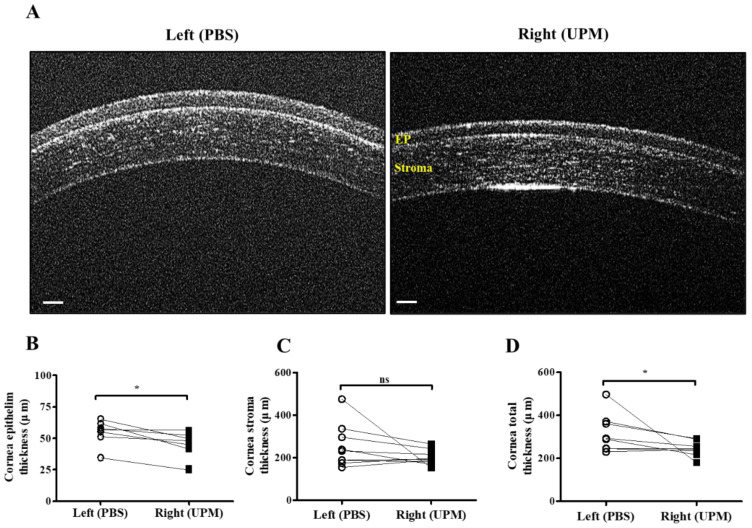
Corneal thickness analysis by SD-OCT at 16 weeks of UPM exposure. (**A**) Images of corneas acquired by SD-OCT are shown. Each layer is marked by yellow letters. The scale bars indicate 100 μm. Direct comparisons of the thickness of the epithelium (**B**), the stroma (**C**) and the total layers (**D**) between the left and right eyes determined by SD-OCT software at 16 weeks are presented. * *p* < 0.05 vs. left eye. EP, epithelium. ns, not significant.

**Figure 5:**
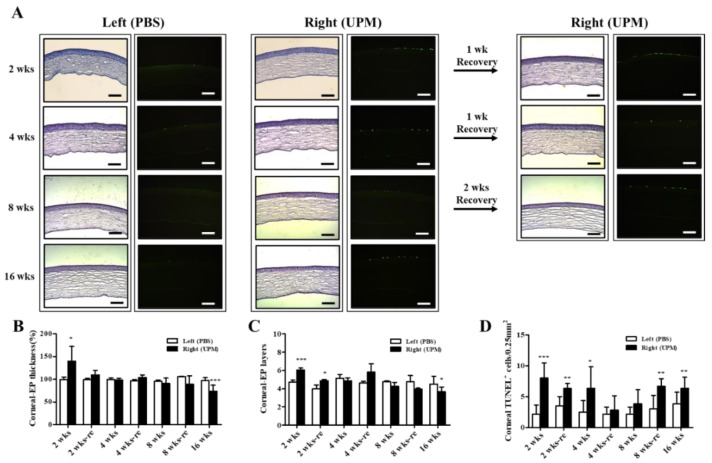
Effect of UPM exposure on corneal epithelial thickness and cell apoptosis according to exposure period. (**A**) Corneal images of hematoxylin and eosin (H&E) and terminal deoxynucleotidyl transferase dUTP nick end labeling (TUNEL) staining acquired at 200× magnification from all experimental periods are shown. The scale bars indicate 100 μm. The graphs of the relative thickness of corneal epithelium (**B**), the number of corneal epithelial layers (**C**) and the counts of apoptotic cells (**D**) are presented. Data are expressed as the mean ± SD. * *p* < 0.05, ** *p* < 0.01, *** *p* < 0.001 vs. left eye at the same time point.

At 16 weeks, the thickness of the epithelium, the stroma and the total layers of the cornea was determined by SD-OCT. The thickness of the corneal epithelium was significantly decreased in the right eye (UPM, 43.5 ± 10.4 μm) compared with the normal left eye (54.5 ± 9.2 μm), whereas the thickness of the corneal stroma was not significantly changed (Figure 4A–C). The total thickness of the cornea was significantly decreased in the right eye (243.7 ± 32.5 μm) compared with the normal left eye (316.5 ± 84.0 μm) (Figure 4D). Thickness and layers of the corneal epithelium and the number of apoptotic cells at all of the time points were determined by histological analysis (Figure 5A). The thickness of the corneal epithelium was significantly increased after 2 weeks of UPM exposure and recovered after rest for one week (Figure 5B). However, the increased thickness was reduced to normal levels at 4 weeks, a decreasing trend was shown at 8 weeks, and the thickness was significantly decreased at 16 weeks. The result of the number of corneal epithelium layers was similar to the thickness results (Figure 5C). Interestingly, apoptotic cells were mainly detected at the outer surface of the cornea, and the number was significantly increased at 2 weeks, which was decreased until 8 weeks and reincreased at 16 weeks (Figure 5D). Periods of rest at 2 and 4 weeks led to a trend of reduced apoptotic cells, whereas no recovery was found at 8 weeks. Therefore, long-term exposure to UPM causes apoptosis of the corneal surface and thinning of the corneal epithelium.

### 2.5. Effect of UPM on Corneal IL-6 Expression

**Figure 6:**
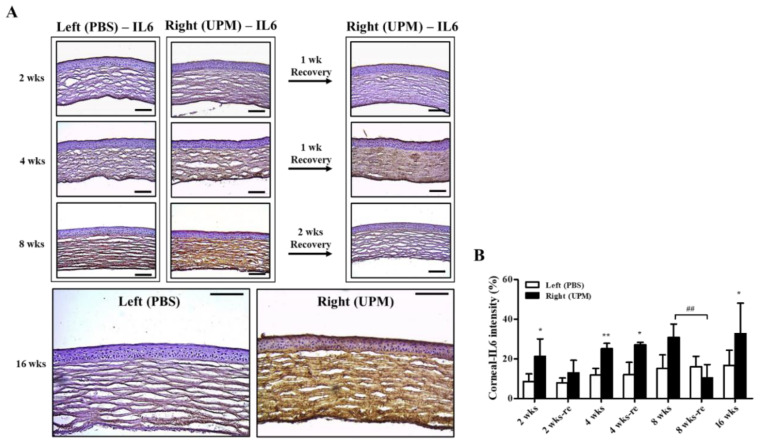
Effect of UPM on corneal IL-6 expression according to exposure period. (**A**) Images of immunohistochemical staining for IL-6 in corneas acquired at 200× magnification from all experimental periods are shown. The scale bars indicate 100 μm. (**B**) The relative intensities of IL-6 in the cornea are expressed as a graph. Data are expressed as the mean ± SD. * *p* < 0.05, ** *p* < 0.01 vs. left eye at the same time point. ## *p* < 0.01 between 8 weeks (8 wks) and two weeks of rest at 8 weeks (8 wks-re) of right eyes.

Corneal damage by PM is mainly induced by increasing reactive oxygen species (ROSs) and inflammatory factors [18,20]. Interleukin-6 (IL-6) is an important factor for corneal inflammation in in vitro dry eye models [21]. The expression and distribution of IL-6 were analyzed by immunohistochemical staining. At 2 weeks, IL-6 was significantly increased in the right eye (21.2 ± 7.2%, left eye: 8.5 ± 3.4%) and constantly increased (32.8 ± 14.4%) during 16 weeks (Figure 6A,B). The period of rest at 2 weeks led to a trend in recovered IL-6 expression, but the period of rest at 4 weeks did not affect IL-6 expression. Interestingly, two weeks of rest at 8 weeks fully reduced IL-6 levels.

### 2.6. Effect of UPM on IOP

As a previous report suggested that PM increased self-reported glaucoma and affected the retinal layer, IOP was measured at 8, 12 and 16 weeks to confirm the effect of long-term exposure of UPM on glaucoma development [19]. The IOP was slightly increased at 8 weeks and consistently increased thereafter (Table 1). At 16 weeks, the ocular pressure on the right eye was 15.7 ± 2.8 mmHg, which was significantly higher than that of the normal eye (13.1 ± 2.2 mmHg).

### 2.7. Effects of UPM on Retinal Thickness and Cell Apoptosis

**Figure 7:**
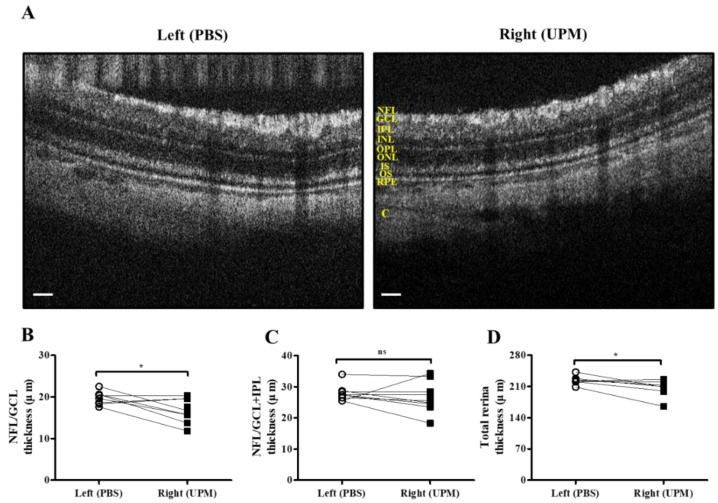
Retinal thickness analysis by SD-OCT at 16 weeks of UPM exposure. (**A**) Images of the retina acquired by SD-OCT are shown. Each layer is marked by yellow letters. The scale bars indicate 100 μm. Direct comparisons between the left and right eye thickness of NFL/GCL (**B**), NFL/GCL + IPL (**C**) and the total layers (**D**) determined by SD-OCT software at 16 weeks are presented. * *p* < 0.05 vs. left eye. NFL, nerve fiber layer; GCL, ganglion cell layer; IPL, inner plexiform layer; INL, inner nuclear layer; OPL, outer plexiform layer, ONL, outer nuclear layer; IS, inner segment; OS, outer segment; RPE, retinal pigment epithelium; C, choroid. ns, not significant.

**Figure 8:**
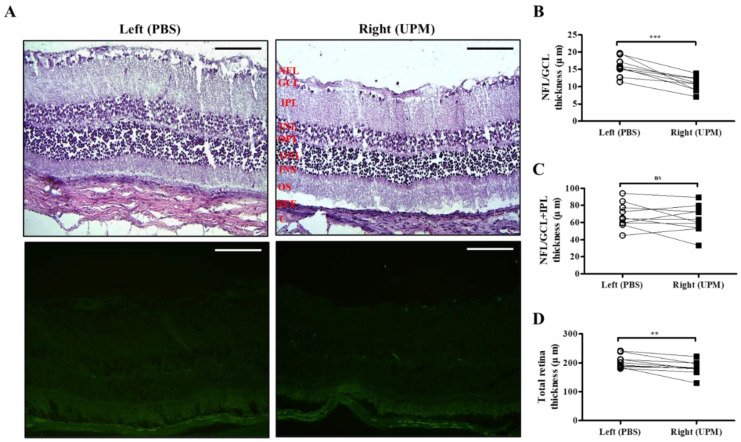
Effect of long-term exposure to UPM on retinal thickness and cell apoptosis. (**A**) The retinal images of H&E and TUNEL staining acquired at 16 weeks are shown. No apoptotic cells were detected by TUNEL staining. The scale bars indicate 100 μm for 200× magnification. Direct comparisons between the left and right eye thickness of NFL/GCL (**B**), NFL/GCL + IPL (**C**) and the total layers (**D**) determined by ImageJ software at 16 weeks are presented. ** *p* < 0.01, *** *p* < 0.001 vs. left eye. NFL, nerve fiber layer; GCL, ganglion cell layer; IPL, inner plexiform layer; INL, inner nuclear layer; OPL, outer plexiform layer, ONL, outer nuclear layer; IS, inner segment; OS, outer segment; RPE, retinal pigment epithelium; C, choroid. ns, not significant.

As shown in Figure 7, retinal images were acquired at 16 weeks by SD-OCT, and the thickness of each layer was determined by OCT Explorer software. The thickness of the nerve fiber layer (NFL)/ganglion cell layer (GCL) was significantly decreased in the right eye (16.7 ± 2.7 μm) compared with the left normal eye (19.8 ± 1.47 μm) (Figure 7A,B). The thickness of the total layers of the retina was also significantly decreased in the right eye (206.0 ± 16.5 μm) compared with the left eye (223.7 ± 9.2 μm), whereas the thickness of the NFL/GCL + inner plexiform layer (IPL) was not different in either eye (Figure 7C,D). As shown in Figure 8, the thickness of retinal layers measured in the histological analysis was similar to the results of the OCT analysis. The thickness of the NFL/GCL was significantly decreased in the right eye (10.7 ± 1.9 μm) compared with the left eye (15.8 ± 2.6 μm) (Figure 8A,B). The thickness of the total layers was also significantly decreased in the right eye (181.7 ± 22.5 μm) compared with the left eye (203.8 ± 20.9 μm), whereas the thickness of NFL/GCL + IPL was not different in either eye (Figure 8C,D). Apoptotic cells in the retina were not detected in either eye (Figure 8A). Therefore, the thickness of the retina was decreased by long-term exposure to UPM, but apoptosis was not detected.

### 2.8. Effect of UPM on Retinal Glial Fibrillary Acidic Protein (GFAP) Expression

**Figure 9:**
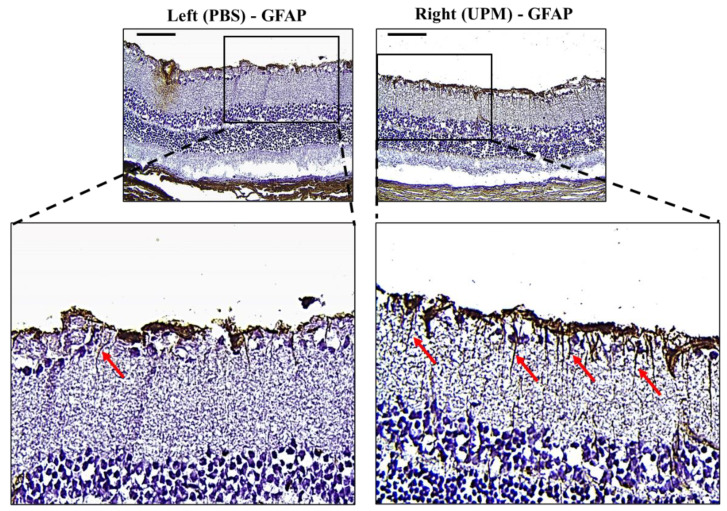
Effect of long-term exposure to UPM on retinal GFAP expression. Images of immunohistochemical staining against GFAP of samples taken from the retinas at 16 weeks. The scale bars indicate 100 μm at 200× magnification. The enlarged image of the right retina showed a sharp and penetrating shape of GFAP from the retinal inner surface (red arrows).

GFAP, an astrocyte marker normally found in the NFL of the retina, is markedly expressed by high IOP [22]. Its expression is well known as a marker of glial activation in response to neural injury [23]. Therefore, the expression and distribution of GFAP were analyzed by immunohistochemical staining. Interestingly, the retina of the right eye from all animals showed increased GFAP expression with long and penetrating shapes from the NFL to the inner nuclear layer (INL) (Figure 9).

### 2.9. Effects of UPM on Conjunctival Goblet Cells

**Figure 10:**
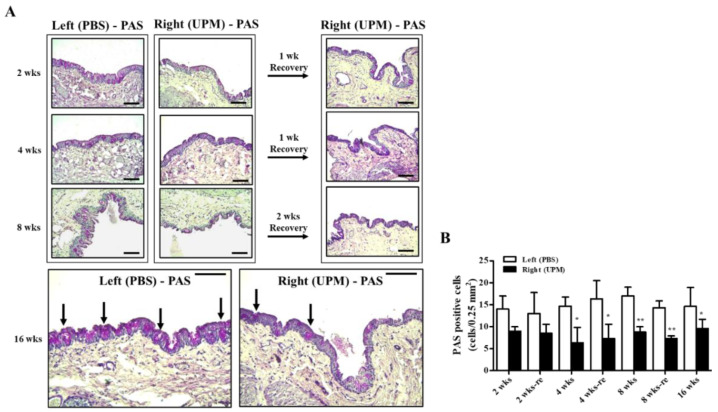
Effect of UPM on conjunctival goblet cells with exposure period. (**A**) The conjunctival images of periodic acid-Schiff (PAS) staining acquired at 200× magnification for all experimental periods are shown. The black arrows indicate PAS-positive cells. The scale bars indicate 100 μm. (**B**) The number of PAS-stained cells is expressed as a graph. Data are expressed as the mean ± SD. * *p* < 0.05, ** *p* < 0.01 vs. left eye at the same time point.

Mucus protein secreted by goblet cells is a component of the tear film and a very important factor in maintaining the stability of tear film [24]. The effects of UPM on conjunctival goblet cells were analyzed by periodic acid-Schiff (PAS) staining (Figure 10A). A decrease in PAS-positive cells was detected in the right eye at all time points during the experiment, although the number was maintained constant (Figure 10B).

## 3. Discussion

This study was designed to elucidate the effects of PM in air pollution on the eye, which is one of the first organs contacting the environment. The effects of UPM for long-term application on various parts of the eye, including the cornea, the conjunctiva and the retina, were carefully investigated. As shown in several human studies, PM is harmful to the ocular surface and causes various discomforts, including presbyopia, instability of tear film, allergic conjunctivitis, toxicity, oxidative stress and inflammation [2,8,25,26]. These symptoms, such as dry eye, have also been reported in animal studies applying various types of PM to the eye [10,11,18]. Some efforts to reduce PM injury by natural products have been investigated and have shown beneficial effects by reducing inflammation and apoptosis [27,28]. However, most animal studies have explored the effects of short-term application and high concentrations of PM. Therefore, the effects of the long-term application of UPM were studied in greater depth in the present study.

The effects of PM have been primarily tested in HCE cells, and increases in ROS, inflammatory factors and autophagy, resulting in decreased migration activity and survival, have been reported [11,15,20]. Similarly, our results showed that both the migration activity and survival of HCE cells were decreased, but the patterns of data differed according to the concentration of UPM (Figure 1). Therefore, a decrease in migration activity by UPM may not be caused by decreased cell survival. It has been predicted that damage induced by PM in the cornea may not be easily recovered. Although this study did not elucidate the detailed mechanisms involved in cell death marker proteins in vitro, UPM was found to significantly decrease cell survival. Therefore, we will undertake follow up studies to determine the cytotoxic effects of UPM on cell death signal-related proteins.

The effects of UPM on the corneal surface were explored by topical application of UPM as a solution to the eyes of rats, and corneal injury was measured at 2, 4, 8 and 16 weeks by fluorescein staining (Figure 2). The fluorescein score was consistently increased during the 16 weeks. The score at 8 weeks did not recover by 2 weeks of rest, although at 2 and 4 weeks, it showed a trend of recovery after 1 week of rest. Therefore, UPM exposure consistently increased corneal damage, which did not spontaneously recover after a certain time. On the other hand, TBUT was decreased at 2 weeks and maintained at similar levels until 16 weeks (Figure 3). The volume of the tear was not changed until 8 weeks and significantly decreased at 16 weeks. As reported in previous studies, damage to the corneal surface and tear instability were exhibited by the short-term application of UPM. However, the damage at 2 weeks in this study was milder than that in previous reports [11,27]. These differences are likely due to differences in animal species and PM sources. Most previous studies have investigated mice, which have smaller ocular surfaces than rats. One group applied the same PM on rats for 2 weeks but used a higher concentration (20 mg/mL). In addition, the WHO guidelines stipulate that PM2.5 does not exceed a 24-h mean of 25 μg/m³ and that PM10 does not exceed a 24-h mean of 50 μg/m³ [29]. A report calculated that the amount of PM to which adults are exposed during light activity is approximately 34.2 μg of PM2.5 (100 μg/m^3^) per hour [15]. It is difficult to compare the PM levels in air exposure and eye drops, but based on the calculation above and considering that the PM solution is easily washable, the rats in this study likely did not receive as severe PM treatment as in other animal studies.

A previous report suggested that PM2.5 application for 2 weeks thickened corneal epithelium through increased inflammatory cell infiltration [2], and another report suggested that fine PM (SRM2786) application for 6 months thinned corneal epithelium through increased ROS and apoptosis [18]. Cellular ROS generation and apoptosis are related to the pathogenesis of dry eye [30,31,32], and thinning of the corneal epithelium has been reported in dry eye patients [33]. Therefore, the time-dependent changes in corneal epithelium by UPM application were analyzed (Figure 4 and Figure 5). The corneal epithelium was thickened by UPM application for 2 weeks and became thinned in a time-dependent manner. The apoptotic cells in the corneal surface also increased for 2 weeks by UPM, but interestingly, they showed a decreasing trend until 8 weeks and reincreased at 16 weeks. The authors of a previous report did not find any evidence of inflammation, cell infiltration or neovascularization in the thinned corneal surface after fine PM application for 6 months [18]. Abnormal morphological changes of corneal epithelial cells or evidence of cell infiltration were also not found in our data, but we revealed that the inflammatory factor IL-6 was constantly increased for 16 weeks (Figure 6). Several studies have investigated corneal thinning in dry eye. Fabiani et al. suggested that excessive apoptosis in epithelium was induced and that not recovered by other homeostatic cycling might lead to epithelial thinning or other damages in severe chronic dry eye [34]. Others have suggested an imbalance between matrix metallopeptidase-1 (MMP-1) and tissue inhibitors of this MMP [35]. This imbalance occurs due to elevated levels of cytokines, which subsequently lead to destructive keratolysis and corneal thinning or even ulceration in severe cases. Corneal thinning in dry eyes increases tear film evaporation or decreases tear turnover, resulting in increased osmolarity of the tear fluid [36]. Hyperosmolarity of tears contributes to increased inflammatory cytokines and MMP-9, which lead to apoptosis of epithelial cells in the cornea and the conjunctiva [37]. The increase in IL-6 in our data is considered to be one of the processes in dry eye and may serve to explain the thinning of the cornea, the increased apoptosis, the reduced tear volume, the increased ocular pressure and the retinal injury. Reduced IL-6 expression upon 2 weeks of rest at 8 weeks was considered to result from temporarily reduced apoptosis; therefore, 2 weeks was considered a sufficient period of rest even though the thickness of the corneal epithelium was not recovered.

A few recent studies have suggested that PM2.5 is associated with self-reported glaucoma and retinal ganglion cell and inner plexiform layer (GCIPL) thickness without IOP changes [19] and that long-term exposure to ambient black carbon increases IOP via oxidative stress [38]. In our data, an increase in ocular pressure was also detected at 8 weeks, and it consistently increased until 16 weeks (Table 1). Increased IOP likely affected retinal damage, although changed levels were small because IOP was detected at low levels under anesthesia [39]. A decrease in the thickness of retinal NFL/GCL, directly affected by IOP change, was detected by SD-OCT and histological analysis (Figure 7 and Figure 8). The thickness between INL to OS are left (191.1 ± 11.3 μm) and right (184.1 ± 5.6) and the difference is not significant. The significance in total thickness is probably produced by accumulated difference of each layers. Therefore, only a result that decrease of NFL/GCL thickness by UPM exposure is clearly proved in this report and the impacts on other layers by UPM exposure should be carefully further studied. GFAP expression, a marker of neural injury in the retina was induced by a high IOP state [22]. In our data, the expression of GFAP was clearly increased, exhibiting long and penetrating shapes in retinal NFL/GCL to IPL (Figure 9), although apoptotic cells were not detected (Figure 10).

Conjunctival goblet cells are also known as an important contributor to the pathogenesis of dry eye by secreting mucins. Mucins play an important role in maintaining the tear film and homeostasis of the ocular surface [40]. Disruption of the tear film can lead to dry eye syndrome [41] and can also induce inflammatory factors, including TNF-α and IL-6 [42]. Here, we analyzed the number of goblet cells by PAS staining, which showed that the number of goblet cells consistently decreased to certain levels compared with normal eyes (Figure 10). This result coincided with the TBUT results, indicating that uniformly lowered conjunctival goblet cells induced tear instability without worsening the condition. The changes in the meibomian gland and lacrimal gland by long-term application of UPM should be further studied. The limitations of this study are the limited number of animals evaluated at each time point except for at the final time point of 16 weeks. However, the data clearly show the changes in each factor of ocular health in a time-dependent manner. These data can serve as a useful basis for investigating the effects of PM on ocular health.

## 4. Materials and Methods

### 4.1. The Source and Components of UPM

We used commercially available UPM (SRM1648a, National Institute of Standards Technology, Gaithersburg, MD, USA). The certificate of analysis of UPM is presented in the supplementary material.

### 4.2. Corneal Epithelial Cell Culture

The simian virus-40 (SV40)-transformed HCE cell line (RIKEN Biosource Center, Tokyo, Japan) was cultured in Dulbecco’s modified Eagle’s medium/Ham’s F-12 (DMEM/F-12) (Welgene, Daegu, Korea) supplemented with 5% fetal bovine serum, 5 µg/mL insulin, 10 ng/mL epidermal growth factor and 0.5% DMSO.

### 4.3. Scratch Wound Healing Assay

The scratch wound healing assay was performed to assess the effect of UPM on the migration of HCE cells, as previously reported [11]. The cells were preincubated with 0–1000 μg/mL UPM suspended in DMEM/F-12 medium without supplements for 24 h. Then, a scratch was induced using a sterile 200 μL pipette tip to make a steady line across the cells on the bottom of the culture dish. Cultural media was replaced with fresh media, and photographs were taken at 0, 3, 6 and 24 h using a microscope equipped with a digital camera (Leica Microsystems, Wetzlar, Germany). The migration rate was calculated by a relatively closed area compared with the area at 0 h, which was measured by ImageJ software (National Institutes of Health, Bethesda, MD, USA). The assay was repeated three times, and each area was measured at least three times.

### 4.4. Cell Viability

The viability of cells treated with different concentrations of UPM was evaluated by a 3-(4,5-dimethylthiazol-2-yl)-2,5-diphenyltetrazolium bromide (MTT) assay. Cells (2 x 10^4^ cells/well) were plated in 96-well plates, and after 24 h, the cells were treated with various concentrations of UPM (1–1000 μg/mL) for 24 h. The cells were incubated with 0.5 mg/mL MTT solution for 2 h, and formazan was dissolved in DMSO. Cell viability was measured by a microplate reader (BioTek, Winooski, VT, USA) at 540 nm.

### 4.5. Animals

**Figure 11:**
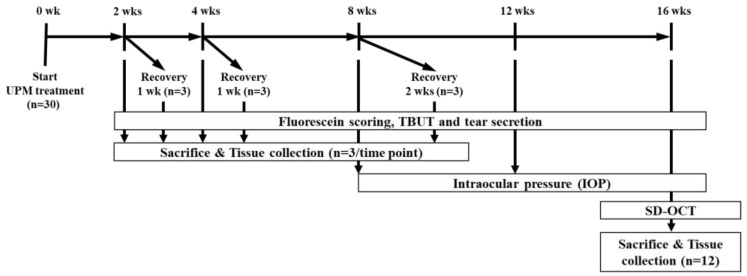
The experimental design for the analysis of the effects of urban particulate matter (UPM) on eyes according to exposure period. All thirty Sprague–Dawley SD rats without abnormalities in both eyes received UPM on the right eye and phosphate buffered saline (PBS) on the left eye. The fluorescein scoring, tear film break-up time (TBUT) and tear volume were assessed at each time point, and then, three rats were sacrificed. Another three rats selected at 2, 4 and 8 weeks underwent periods of rest for 1 or 2 weeks, and the above analysis was performed before sacrifice. Ocular pressure was measured at 8, 12 and 16 weeks. Spectral domain optical coherence tomography (SD-OCT) analysis was performed at 16 weeks.

Five-week-old male Sprague-Dawley (SD) rats were purchased from Semtaco Animal, Inc. All the experimental procedures were conducted in accordance with the relevant guidelines for the care of experimental animals and approved by the Institutional Animal Care and Use Committee (IACUC) of the Bioresources and Technology (B&Tech, Gwangju, Korea) Co., Ltd., Republic of Korea (B&Tech, Gwangju, Korea) (approval number: BT-001-2019, 10 July 2019). Animals were quarantined before the experiment and adapted to the environment for 1 week. All included rats had normal ocular surfaces of the eyes, as observed under a stereomicroscope. UPM (SRM 1648a, NIST, Gaithersburg, MD, USA) was dissolved in phosphate buffered saline (PBS) to prepare a 5 mg/mL eye drop solution. The solution was stored in glass bottles at 4°C and used within one week. All rats received 10 μL of UPM solution onto the right eye four times per day. The left eye of all animals received 10 μL of PBS and was used as a normal control. During the total period, the three ocular tests (fluorescein staining, TBUT and the phenol red thread tear test (PRT-TEST)) were performed on all animals at 2, 4, 8 and 16 weeks to test the effects of UPM on the eye (Figure 11). Additionally, three rats were selected at each time point to undergo a rest period for one or two weeks. Then, the same tests were performed to confirm the recovery from ocular damage induced by UPM. Three animals at each time point were sacrificed after the three ocular tests, and tissues including the eyeball and the eyelid on both sides were collected for further analysis. Ocular pressure was additionally analyzed at 8, 12 and 16 weeks, and OCT analysis of the cornea and the retina was performed at the final 16-week time point one day before sacrifice.

### 4.6. Tear Volume Measurement

The examination technique and the environment were kept as consistent as possible. All rats were anesthetized with an intraperitoneal injection of ketamine (50 mg/kg) and xylazine (5 mg/kg) mixture before the measurements. The phenol red thread tear test was performed as previously described [43]. Briefly, the tear volumes of each rat were measured using the phenol red thread (Jingming Ltd., Tianjin, China) test (PRT-test). One end of the thread was hung on the lower lateral 1/3 of the eyelid for 1 min. The tear volume was measured as the length of the thread that turned red due to tear fluid and was expressed in millimeters (mm).

### 4.7. Fluorescein Staining and TBUT

Fluorescein staining and TBUT were performed as previously described [11]. Briefly, 2 μL of 0.5% sodium fluorescein was dropped into the conjunctival sac and the eyelid was manually closed three times, then the TBUT was recorded using a stereomicroscope with a cobalt blue filter. The time was recorded, repeated three times and the average value was obtained. After 90 s, the fluorescein-stained corneal surface was observed under a stereomicroscope, and images were captured by a CMOS camera (Jenoptik AG, Jena, Germany). The images at 16 weeks were specifically captured by a funduscope with an integrated fiber optic light source equipped with a low light fluorescence camera (OcuScience, Henderson, NV, USA). Corneal injury was scored as follows: 0, no spot; 1, ≤30 separate spots; 2, ≥30 separate spots in some area; 3, many separate spots without a closed spot in the overall area; 4, many separate spots with closed spots in the overall area.

### 4.8. Intraocular Pressure (IOP)

IOP was measured using a TONOLAB tonometer (Icare Finland Oy, Vantaa, Finland) according to the manufacturer’s recommended procedures. Briefly, each rat was anesthetized by isoflurane before measuring IOP, and a 1-min period with the minimum isoflurane delivery was employed to stabilize IOP. Then, the mean of five valid IOP readings made by a tonometer was obtained from each eye at the center of the cornea.

### 4.9. Spectral Domain Optical Coherence Tomography (SD-OCT)

Spectral domain optical coherence tomography (SD-OCT) was performed to analyze the thickness of the corneal epithelium and the retinal layers in both eyes of rats at 16 weeks of UPM treatment. Rats were anesthetized with intraperitoneal injection of a ketamine (50 mg/kg) and xylazine (5 mg/kg) mixture, and the pupils were dilated with isopto-atropine (Alcon, Fort Worth, TX, USA). Then, the rats were placed on an imaging stage in front of the OCT imaging device (OcuScience, Henderson, NV, USA), and the cornea or the retina was visualized by contacting the OCT lens to the gel. For corneal thickness analysis, four images of the corneal surface were captured at the same time by B-scan with 3-μm axial resolution centered on the cornea, and an average image was used. The thickness of the corneal epithelium, the stroma and the total layers were measured by iVivo Ophthalmic OCT software (OcuScience, Henderson, NV, USA). For retinal analysis, average images were captured by a horizontal B-scan with a 3-μm axial resolution, and volume scan images were acquired by 128 B-scans per volume centered on the optic disc. Retinal layers were evaluated by OCT Explorer software (OCT Explorer, Retinal Image Analysis Lab, Iowa Institute for Biomedical Imaging, Iowa City, IA, USA, Software V.3.8.0), and the nerve fiber layer/ganglion cell layer (NFL/GCL), NFL/GCL + IPL and the total retinal thickness were calculated.

### 4.10. PAS and Hematoxylin and Eosin (H&E) Staining

Eyes and eyelids were fixed for 48 h in 10% buffered formalin. Then, the eyes were coronally bisected to separate the cornea and the retinal region. All tissues were immersed in 30% sucrose solution and embedded in OCT compound. Tissue sections of 5-μm thickness were stained with PAS (ScyTek, Logan, UT, USA) or H&E. Goblet cell density was determined by counting PAS-positive cells in three different sections of each animal and taking the average. The thickness of the cornea and the retina was measured five times in the same position of each section of the animal by ImageJ software (National Institutes of Health, Bethesda, MD, USA), and the average was used.

### 4.11. Terminal Deoxynucleotidyl Transferase dUTP Nick end Labeling (TUNEL) Staining

Apoptotic cells were determined by an In Situ Cell Death Detection kit (Sigma, St Louis, MO, USA) according to the manufacturer’s recommended procedures. The nuclei were counterstained by mounting medium containing DAPI. Apoptotic cells were observed under a fluorescence microscope. The number of apoptotic cells was counted in three different regions of each section, and the average was taken.

### 4.12. Immunohistochemical Staining

Sections were stained with antibodies against interleukin-6 (IL-6) (Abcam, Cambridge, MA, USA) or GFAP (Santa Cruz, CA, USA). Subsequently, sections were incubated with a detection kit (Dako, Carpinteria, CA, USA) including secondary antibody and 3,3’-diaminobenzidine tetrahydrochloride (DAB)/substrate solution according to the manufacturer’s recommended procedures. Nuclei were counterstained by hematoxylin. Images were obtained using an Olympus BX50 microscope (Olympus, Tokyo, Japan).

### 4.13. Statistical Analysis

Data are presented as the mean ± standard deviation (SD). The data were statistically evaluated using Student’s *t*-test or two-way analysis of variance (ANOVA) with GraphPad Prism 5 version 5.01 for Windows (GraphPad, Inc., San Diego, CA, USA) software programs. Statistical significance was indicated when *p* < 0.05.

## 5. Conclusions

There are several pieces of evidence reported in human and animal studies that suggested that particulate matter in air pollution is the major component to induce ocular diseases. However, most of the studies are investigated in vitro or focused on the short-term effects. In this study, we carefully explored time-dependent ocular changes various parts of eyes and revealed that topical long-term application of UPM induces not only dry eye-like changes, including corneal injury, corneal thinning, tear instability and reduced tear volume, but also retinal injury and structural changes. Especially the results that long-term exposure to UPM increases ocular pressure and induces both neuronal injury and retinal NFL/GCL thinning provide new insight for investigating the effects of particulate matter on eyes. This study suggests that the harmful effects of PM on the eye, which are not detected by short-term exposure, should be considered in investigating mechanisms and therapeutics.

## Figures and Tables

**Table 1:** Effect of long-term exposure to UPM on ocular pressure.

	Ocular Pressure (mmHg)		
	Left (PBS)	Right (UPM)	*p*-Value
8 weeks	12.9 ± 1.3	13.5 ± 1.4	0.239 ^ns^
12 weeks	12.9 ± 1.8	14.0 ± 1.7	0.031 *
16 weeks	13.1 ± 2.2	15.7 ± 2.8	0.003 **

Data are expressed as the mean ± SD. * *p* < 0.05, ** *p* < 0.01 vs. left eye at the same time point. ns, not significant.

## Supplementary Materials

Supplementary materials can be found at https://www.mdpi.com/1422-0067/21/14/4976/s1.
